# Precision Hard Turning of Ti6Al4V Using Polycrystalline Diamond Inserts: Surface Quality, Cutting Temperature and Productivity in Conventional and High-Speed Machining

**DOI:** 10.3390/ma13245677

**Published:** 2020-12-12

**Authors:** Elshaimaa Abdelnasser, Azza Barakat, Samar Elsanabary, Ahmed Nassef, Ahmed Elkaseer

**Affiliations:** 1 Department of Production Engineering and Mechanical Design, Port Said University, Port Fuad 42526, Egypt; alshymaa.gamal@eng.psu.edu.eg (E.A.); samar.abaas@eng.psu.edu.eg (S.E.); nassef12@eng.psu.edu.eg (A.N.); 2 Mechanical Engineering Department, Faculty of Engineering, Helwan University, Cairo 11792, Egypt; barakatazza@h-eng.helwan.edu.eg; 3 Institute for Automation and Applied Informatics, Karlsruhe Institute of Technology, 76344 Karlsruhe, Germany

**Keywords:** precision hard turning, Ti6Al4V, PCD inserts, surface quality, productivity, cutting temperature, conventional machining, high-speed turning

## Abstract

This article presents the results of an experimental investigation into the machinability of Ti6Al4V alloy during hard turning, including both conventional and high-speed machining, using polycrystalline diamond (PCD) inserts. A central composite design of experiment procedure was followed to examine the effects of variable process parameters; feed rate, cutting speed and depth of cut (each at five levels) and their interaction effects on surface roughness and cutting temperature as process responses. The results revealed that cutting temperature increased with increasing cutting speed and decreasing feed rate in both conventional and high-speed machining. It was found that high-speed machining showed an average increase in cutting temperature of 65% compared with conventional machining. Nevertheless, high-speed machining showed better performance in terms of lower surface roughness despite using higher feed rates compared to conventional machining. High-speed machining of Ti6Al4V showed an improvement in surface roughness of 11% compared with conventional machining, with a 207% increase in metal removal rate (MRR) which offered the opportunity to increase productivity. Finally, an inverse relationship was verified between generated cutting temperature and surface roughness. This was attributed mainly to the high cutting temperature generated, softening, and decreasing strength of the material in the vicinity of the cutting zone which in turn enabled smoother machining and reduced surface roughness.

## 1. Introduction

Titanium-based alloys are attracting considerable attention in a wide range of industries due to their unique mechanical and physical properties [1]. In particular, they have gained popularity in aircraft engine and airframe manufacture as they offer a strength to weight ratio higher than aluminum or steel, in addition to which they offer excellent corrosion and creep resistance [2,3]. Titanium alloys are widely used in industries where corrosion resistance is important, such as the marine industry to resist seawater corrosion, for components in chemical processing equipment and, increasingly important, in the biomedical industry for prosthetic joints and implants where their low elastic modulus and high strength are added advantages [4,5].

For aerospace components, machining is an important manufacturing process for superalloys such as titanium alloys, to achieve the required tight tolerances and complex geometries of the produced parts [6]. However, machining of titanium-based alloys has proved to be problematic and it is difficult to simultaneously attain more accurate components, lower machining costs, reduced material wastage, and higher rates of production [7]. The main factors for the low machinability of titanium alloys are their low thermal conductivity [8], high chemical reactivity [9], and low modulus of elasticity [10] all of which affect machining accuracy, damage to the cutting tool, surface finish and surface quality [1].

Surface integrity is the most important factor quantifying the quality and performance of the machining process [11,12]. Surface quality is correlated with length of the component’s service life as poor surface finishing can lead to early failure of the machined products due to friction between parts and consequent fatigue effects [13,14]. Thus, numerous researchers have studied the machining factors that affect the surface integrity of machined titanium-based alloys in order to improve the surface quality. Studies of the surface integrity of titanium alloys have included the effects on measured surface roughness [15], residual stress [16] and microhardness [17] of varying cutting factors such as process parameters and tool geometry. The effect of these factors on cutting force [18], temperatures generated [19], and friction between the tool and target material [20] have also been investigated as all influence surface quality. Surface roughness is the most important factor when measuring surface integrity due to its determining influence on material service life—high levels of surface roughness lead to rapid wear of the machined surface and increase the risk of cracks and corrosion due to surface irregularities [13]. Therefore, there is a real need to optimize machining factors [21,22] and develop techniques [23] to improve surface roughness when machining titanium alloys.

Many studies have examined the influence of process parameters and cutting tool geometries on surface roughness of titanium-based alloys. The nose radius of the cutting tool and the feed rate were found to be the most significant parameters determining surface roughness followed by cutting speed and depth of cut [24,25]. A lower feed rate and larger nose radius result in a smoother surface [26]. Coolant and lubrication were found to be effective methods for reducing surface roughness and improving surface quality [27,28]. However, some researchers reported that the type of coolant used had little effect on obtainable surface roughness [8].

The very high cutting temperature generated during machining titanium-based alloys is one reason for their poor machinability [29]. The low thermal conductivity of titanium alloys prevents the dissipation of heat generated during cutting, resulting in the very high temperature of the cutting zone which causes damage to the machined surface and cutting tool [30]. Cutting temperature generated during machining is significantly influenced by cutting conditions [31,32] and cutting tool materials and geometry [33,34]. Various studies have been conducted in order to reduce the temperature during the machining of titanium alloys, and these have been based on optimizing cutting conditions or used methods such as vibration assisted machining [35] which showed a good result in reducing temperature of cutting, using a rotary tool [36], using lubrication to reduce friction between the tool and target material [37] or using emerging cooling systems [38].

Although there are benefits from using coolants and lubrication during machining in terms of chip evacuation [39], reduced thermal distortion and tool wear [40], minimum quantity lubrication (MQL) and dry machining are preferred these days as they are more environmentally friendly and reduce the overall machining costs [41,42]. Dry machining has the benefit of decreasing power consumption because the high cutting temperature generated can help by slightly softening the target material in the pre-cut area, reducing its hardness and the required cutting force. This makes shearing of the material easier which is especially important when machining hard-to-cut materials [43]. For this reason, high-speed machining (HSM) has been found to have benefits when machining hard-to-cut materials [44].

In HSM, increasing cutting speed leads to a higher cutting temperature [45] which softens the target material and reduces the cutting force [43]. If this high temperature is controlled well, it can result in higher surface quality, lower stress, burr-free edges of parts and increase productivity [45]. Moreover, HSM could help minimize thermal effects as a large proportion of the heat generated is removed by chips ejected from the cutting zone, which can reduce tool wear and increase tool life [46]. Although many materials such as aluminum and stainless steel show excellent performance when subject to HSM, the application of HSM to titanium alloys is still a challenge [47,48]. This is due to low thermal conductivity of titanium alloys, which means a high temperature at the tool–chip interface that can accelerate tool wear and negatively affect tool life [49,50,51].

Excessive tool wear rates when machining titanium alloys is one of the reasons they are difficult-to-cut materials [52,53], so proper selection of a suitable cutting tool is necessary for machining reliability and high productivity [54]. Cutting tool materials which remain hard at high temperatures, with high thermal conductivity and high wear resistance are preferred when machining hard-to-cut materials [55]. Researchers have studied the machining of titanium-based alloys with different cutting tool materials with different coatings [7,56] and it has been demonstrated that polycrystalline diamond (PCD) tools show excellent performance in machining titanium [57]. That is due to their high thermal conductivity [58] which means good heat dissipation from the cutting zone during cutting. Diamond is the hardest cutting tool material available to the machining industry, and PCD has a low coefficient of friction giving diamond tools better wear resistance compared to other tool materials [30]. In practice, PCD tools showed better machining performance when compared to other cutting tool materials, and longer tool life [59], and can operate at higher values of the cutting parameters [44,60].

Numerous previous research studies have investigated the machining of titanium alloys in terms of cutting force, surface integrity, cutting temperature and tool wear. However, to the best of the author’s knowledge, the body of reported research is largely limited to investigations of the effects of process parameters on a single response. In the relatively few investigations that have examined cutting parameters and the simultaneous variation of more than one process responses, there are no reports which examined their inter-correlation or quantified their interaction. In particular, there has been a lack of published studies that report how changes in a range of process parameters interact to affect resultant surface roughness, heat generated and their dependent relationships. In this context, this paper reports an experimental investigation into the effect of the interaction of a range of cutting parameters and conditions (including conventional and HSM) on cutting temperature and generated surface roughness. This paper includes an explanation of the mechanisms, correlating cutting temperature and resultant surface quality when using PCD tools to machine Ti6Al4V under the wide range of cutting parameters. In addition, the paper assesses how the applied process parameters impact on material removal rate (MRR) and thus productivity for both conventional and HSM.

## 2. Experimental Work

### 2.1. Materials and Experimental Setup

Turning tests were carried out on a computer numerical control (CNC) lathe (PLOY GIM-42) (5.5 kW spindle motor, 6000 rpm maximum spindle speed). All experiments were conducted under dry machining for the benefits previously explained, using PCD inserts due to their being less affected by high cutting temperature than other tool materials [60]. The PCD inserts had a 0° rake angle, 11° clearance angle, and 1.2 mm nose radius. The tool holder (PDJNR 2020 K15) was by TaeguTec (Daegu, Korea). All experiments were conducted for a length of cut of 30 mm.

Cylindrical Ti6Al4V specimens, 25 mm diameter were used as workpieces. Material hardness was measured by a Vickers test (Indentec, Brierley Hill, UK) (367 V_30_). Chemical composition of the target material was determined, and the results are shown in Table 1. Mechanical and physical properties of Ti6Al4V were reported in [61], and found to have a tensile strength of 931 MPa, 862 MPa Yield strength, thermal conductivity of 7.3 W/m K at 20 °C and specific heat of 709 J/kg K for the range of temperature between 20 to 100 °C.

### 2.2. Process Parameters and Design of Experiments

The experiments were performed by turning the Ti6Al4V workpiece with different levels of three process parameters: cutting speed, feed rate and depth of cut. Cutting performance for each set of process parameters was determined in terms of surface roughness, cutting temperature and MRR. The parameter ranges were selected based on pre-experiments carried out to test chip control and surface roughness. This was due to the difficulty of chip evacuation when turning titanium because the continuous chip produced can tangle around the workpiece and negatively affect the accuracy of the results. Two different experimental processes based on ranges of cutting speed, feed rate and depth of cut were used:

- High-speed machining [44], which has higher levels of cutting speed and feed rate.
- Conventional machining [44] with lower levels of cutting speed and feed rate.

Table 2 and Table 3 show the levels, and equivalent normalized values, of cutting speed, feed rate and depth of cut used in the case of HSM and conventional machining, respectively.

A central composite method was used to design the experimental trials. This method enables the study of three factors at five levels with a minimum number of experiments. The design of experiments used for the above two cases can be seen in Table 4.

### 2.3. Characterization

**Figure 1:**
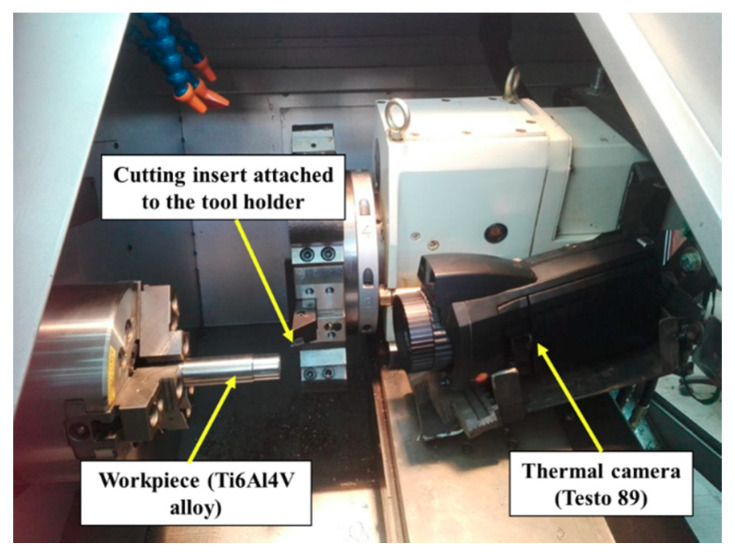
Experimental test rig: a CNC lathe with thermal imager attached.

A thermal imager (testo 890) with a resolution of 640 × 480 pixels and thermal sensitivity <40° mK was used to measure cutting temperature during machining with a measuring range up to 1200 °C. This provided an image of the temperature distribution of the cutting process with the imager fixed along the centre line of the workpiece and opposite the free end of the workpiece, as shown in Figure 1.

A Surtronic (3 stylus) profilometer (Taylor Hobson, Leicester, UK) was used to measure surface roughness of specimens. The cutoff length used was 0.8 mm, and 10 measurements of surface roughness were taken for each specimen and the average value determined.

### 2.4. Regression Modelling

Statistical regression using a quadratic expression, Equation (1), was used to define the relationship between experimentally measured response and input process parameter.

(1) $$y=b_{0}+\sum b_{i}x_{i}+\sum b_{ii}x_{ii}^{2}+\sum b_{ij}x_{i}x_{j}$$

where ‘*y*’ is the response (e.g., cutting temperature in °C or surface roughness, Ra in µm), ‘*b*_0_’,*’b_i_*’, ‘*b_ii_*’ and ‘*b_ij_*’ are the regression coefficients, and ‘*x_i_*’ and ‘*x_j_*’ are the *i*th and *j*th values of the input parameter, respectively.

To develop a robust quadratic regression model for three process parameter inputs (cutting speed, feed rate, and depth of cut) requires 10 terms. Equations (2)–(5), show, respectively, the predicted values of cutting temperature (in °C) and surface roughness (in µm) for conventional speed cutting and high-speed cutting of the Ti6Al4V specimens. In the equations, ‘*v_cn_*’, ‘*f_n_*’, and ‘*a_pn_*’, are the normalized values of cutting speed, feed rate, and depth of cut. The measured values of the process parameters were normalized to [−1,1].

In Equation (2), ‘*T_conventional_*’ is the predicted cutting temperature for conventional cutting; R-squared was 0.972, and adjusted R-squared was 0.952.

(2) $$\begin{array}{l} T_{conventional}=327.240-32.359\;f_{n}+12.157\;{v_{c}}_{n}+10.452\;{a_{p}}_{n}-16.877\;f_{n}{v_{c}}_{n}\\ \;+3.232\;f_{n}{a_{p}}_{n}+0.359\;{v_{c}}_{n}{a_{p}}_{n}-23.778\;f_{n}^{2}+39.722\;{v_{c}}_{n}^{2}\\ \;-4.278\;{a_{p}}_{n}^{2} \end{array}$$

‘*T_high-speed_*’ is the predicted cutting temperature for high-speed cutting; R-squared was 0.931, and adjusted R-squared was 0.879.

(3) $$\begin{array}{l} T_{high\text{-}speed}=568.580-68.569\;f_{n}+95.446\;{v_{c}}_{n}+23.932\;{a_{p}}_{n}\\ \;+39.859\;f_{n}{v_{c}}_{n}-3.950\;f_{n}{a_{p}}_{n}+0.359\;{v_{c}}_{n}{a_{p}}_{n}-46.840\;f_{n}^{2}\\ \;-60.340\;{v_{c}}_{n}^{2}-5.840\;{a_{p}}_{n}^{2} \end{array}$$

‘*Ra_conventional_*’ is the predicted average roughness with conventional machining; R-squared was 0.831, and adjusted R-squared was 0.705.

(4) $$\begin{array}{l} Ra_{conventional}=0.820+0.266\;f_{n}-0.169\;{v_{c}}_{n}+0.049\;a_{p}-0.040\;f_{n}{v_{c}}_{n}\\ \;-0.032\;f_{n}a_{p}+0.018\;{v_{c}}_{n}a_{p}+0.105\;f_{n}^{2}+0.010\;{v_{c}}_{n}^{2}+0.270\;{a_{p}}_{n}^{2} \end{array}$$

‘*Ra_high-speed_*’ is the predicted average roughness with high-speed machining; R-squared was 0.930, and adjusted R-squared was 0.877.

(5) $$\begin{array}{l} Ra_{high\text{-}speed}=0.751+0.446\;f_{n}-0.141\;{v_{c}}_{n}-0.110\;{a_{p}}_{n}-0.100\;f_{n}{v_{c}}_{n}-0.079\;f_{n}a_{p}\\ \;-0.187\;{v_{c}}_{n}{a_{p}}_{n}+0.122\;f_{n}^{2}+0.187\;{v_{c}}_{n}^{2}\\ \;-0.108\;{a_{p}}_{n}^{2} \end{array}$$

These regression models (Equations (2)–(5)) were then used to analyze the effects of the different input parameters, singly and in combination, on the measured cutting temperatures and surface roughnesses. These are described below. In addition, analysis of variance (ANOVA) was used to determine the input parameters with greatest impact on the responses.

## 3. Results and Discussion

### 3.1. Cutting Temperature

**Figure 2:**
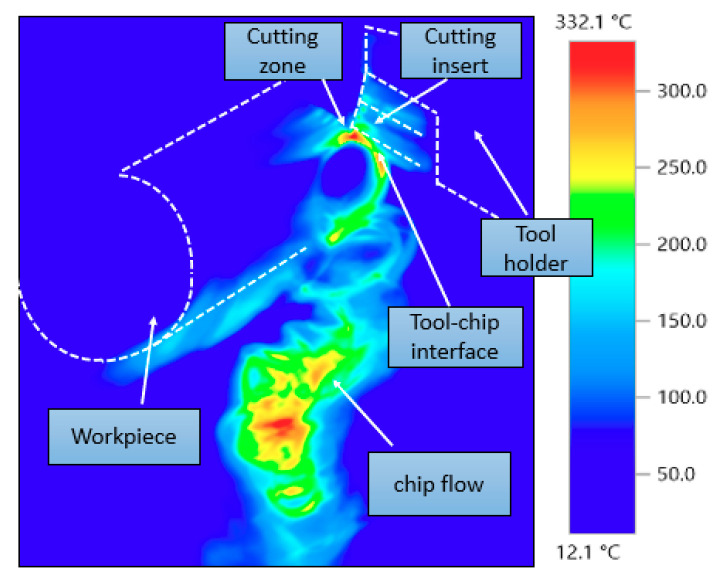
Cutting temperature distribution by thermal imager at at *f* = 0.15 mm/rev, *v_c_* = 100 m/min and *a_p_* = 0.2 mm.

For all experiments the temperature was measured at the end of the cutting stroke. Figure 2 shows the result given by the thermal imager for one of the experiments for temperature distribution, during the machining process, for processed material, chips and insert. A great difference between the temperature generated in the cutting zone area and the temperature of the surrounding area in the target material can be seen. The maximum temperature was in the cutting zone and chips produced, while the surrounded area of the workpiece had a relatively low temperature. This reflects the low thermal conductivity of the titanium alloy which retained the heat in the cutting zone area and prevented dissipation of the heat generated during cutting. The results agree with [62], where the authors used an infrared camera to measure the temperature distribution when machining Ti6Al4V. It was reported that the high temperature generated due to cutting did not penetrate deep into the machined material and most of the heat was carried away by the chips. Distribution of temperature on the cutting insert showed a moderate temperature with uniform distribution over the insert and surrounding area, confirming good heat dissipation by PCD tools.

**Figure 3:**
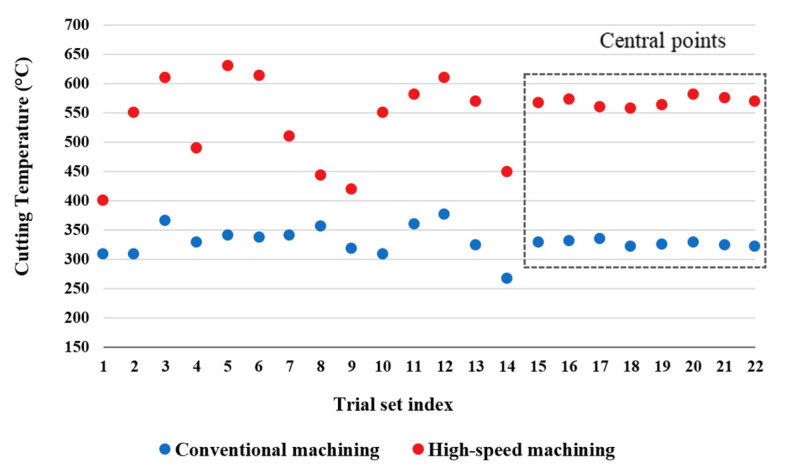
Results of measured cutting temperature for twenty-two individual trials for conventional and high-speed machining.

Figure 3 shows the measured values of cutting temperature for conventional machining and HSM. The results, especially low noise/variation of the results obtained for the central points, refer to a stable process which increased the possibility for developing a process model using these experimental results.

The results showed a considerable difference in temperature between conventional machining and HSM. For all trials, the recorded cutting temperature was higher for HSM than conventional machining. The temperature increased by 65% on average in the case of HSM compared to conventional machining, 544 °C compared to 330 °C.

**Figure 4:**
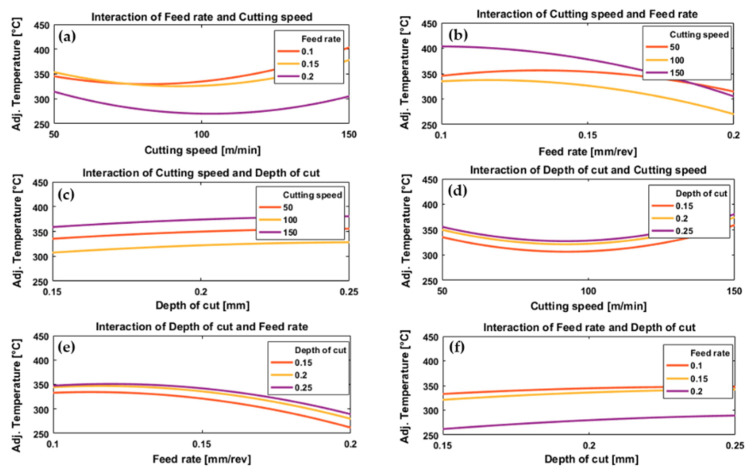
Plots depicting the interactive effects of feed rate, cutting speed, and depth of cut on cutting temperature of Ti6Al4V specimens by conventional machining; (**a**) the effect of cutting speed at different levels of feed rates, (**b**) the effect of feed rate under different levels of cutting speeds, (**c**) the effect of depth of cut at different levels of cutting speeds, (**d**) the effect of cutting speed under different levels of depth of cuts, (**e**) the effect of feed rate at different levels of depth of cuts and (**f**) the effect of depth of cut under different feed rates.

**Figure 5:**
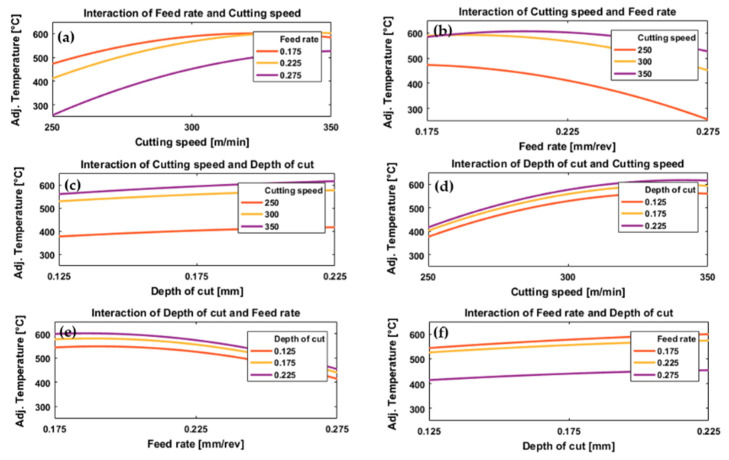
Plots depicting the interactive effects of feed rate, cutting speed, and depth of cut on cutting temperature of Ti6Al4V specimens by high-speed machining; (**a**) the effect of cutting speed at different levels of feed rates, (**b**) the effect of feed rate under different levels of cutting speeds, (**c**) the effect of depth of cut at different levels of cutting speeds, (**d**) the effect of cutting speed under different levels of depth of cuts, (**e**) the effect of feed rate at different levels of depth of cuts and (**f**) the effect of depth of cut under different feed rates.

Figure 4 and Figure 5 illustrate the effect of process parameters (feed rate, cutting speed and depth of cut) and their interactions on measured cutting temperature when machining Ti6Al4V by conventional machining and HSM, respectively. These graphs were plotted using the regression models, Equations (2) and (3), established for predicting cutting temperature for conventional and high-speed machining, respectively.

In the case of conventional machining, the results showed a gradual decrease in cutting temperature with increasing cutting speed from 50 to about 100 m/min, then the temperature increases with increasing cutting speed from about 100 to 150 m/min for all values of feed rate and depth of cut, see Figure 4a,d. Increasing temperature with increased cutting speeds from 100 to 150 m/min correlates with a high MRR due to an increase in the rate of energy dissipation through plastic deformation and friction [63]. Therefore, increasing the cutting speed can increase the temperature of the cutting zone. These results agree with those presented in [31]. At low cutting speeds (50 m/min), an increase in cutting temperature was also observed, possibly due to increased resistance forces and stresses at the tip of the cutting tool which increased the coefficient of friction for tool–chip contact [64,65] and, as a result, the tool–chip interface temperature increased. Increasing cutting speed from 50 to 100 m/min, Figure 4a,d reduced the temperature. This was due to reduced tool–chip contact length and coefficient of friction, and this led to a decrease in heat generated by friction between the tool and the workpiece. Therefore, as cutting speed increased from 50 m/min we first saw a drop and then an increase in temperature.

Different trends were observed for the effect of cutting speed on cutting temperature for HSM, see Figure 5a,d. For all feed rates and depths of cut there was a relatively rapid increase in temperature with an increase in cutting speed, although this tended to level off at higher speeds, especially for the lower feed rates, Figure 5a. The levelling off at the highest cutting speeds, see Figure 5a,d may be due to the relatively short machining time due to the small length machined which resulted in a decreased time of contact between tool and workpiece, decreasing the total heat generated which could have reduced cutting temperature [33].

With regard to the effect of feed rate on cutting temperature, see Figure 4b,e and Figure 5b,e, there was generally an inverse relationship between cutting temperature and feed rate for all values of cutting speed and depth of cut for both conventional and HSM. For low values of feed rate, the cutting tool was in contact with the workpiece for longer which leads to increased temperature of the tool-chip interface [33] and the low thermal conductivity of the titanium alloy prevented the dissipation of the heat generated which maintained the temperature on the tool rake face. Additionally, an increase in the coefficient of friction [65] at low feed rates resulted in more heat generation. These results agree with [66], where the authors used PCD inserts to cut titanium alloy for a range of feed rates starting at 0.1 mm/rev up to 0.15 mm/rev. The authors reported that temperature decreased with increasing feed rate. They suggested the reason was increasing chip thickness at higher feed rates which takes more heat away and changes the heat distribution at the cutting zone. However, as would be expected, the result differs from studies based on machining titanium alloys using other cutting tool materials [30].

Looking at Figure 4a,b, we see that for the interaction between cutting speed and feed rate in the case of conventional machining, the feed rate has only a small effect on cutting temperature at low cutting speeds, with a greater variation of cutting temperature with feed rate at higher cutting speeds. The reduction in temperature by interaction of higher cutting speed and higher feed rates is due to reduced machining time which reduces the temperature generated by the cutting process. Moreover, at higher cutting speed there is a softening of material by the high temperature generated which decreases the cutting force associated with higher feed rates [18].

With HSM we see in Figure 5a,b that for a given feed rate temperature increases with cutting speed for all values of feed rate tested, but that at high cutting speeds temperature varied only slightly with feed rate. However, for low cutting speeds temperature tended to fall as feed rate increased. The latter effect is due the reduced machining time between the tool and workpiece. Note that the feed rate range chosen in the case of HSM was greater than the range of feed rate chosen for the conventional case.

Depth of cut had a significant effect on cutting temperature for all values of feed rate and depth of cut in both conventional and HSM, Figure 4c,f and Figure 5c,f. With increasing depth of cut, the cutting temperature increased in both cases. Increasing depth of cut requires an increase in cutting force [18] and more energy will be dissipated in the cutting process as more material is removed by the workpiece, with higher MRRs and higher cutting temperatures. These results agree with [66].

Figure 4c shows the interaction between cutting speed and depth of cut for conventional machining, and it was found that for all cutting speeds tested the temperature increased with depth of cut. With high-speed machining, Figure 5c, the increase in temperature with increase in depth of cut was much less pronounced than for conventional machining. This can be attributed to the softening effect of pre-cut material occurred due to higher temperature generated in the case of high-speed machining, which resulted in a reduction in the specific cutting resistance of the processed material, and thus a change in applied depth of cut is associated with less variation in cutting energy and generated temperature. This is not the case for conventional machining in which there is no pre-cut softening neither reduction in the specific cutting energy and consequently a more dominant effect of the depth of cut can be observed when compared with the case of high-speed machining. In conventional machining, Figure 4d, the graph of temperature against cutting speed is a U-shaped curve, dipping to a minimum in the region of 90 m/min for all depths of cut tested. The curves of temperature against cutting speed were parallel, with higher temperatures generated by deeper cuts. In HSM, Figure 5d, depth of cut had little effect on cutting temperature at low cutting speed (250 m/min) but the effect grew slightly with increasing cutting speed.

In conventional machining, Figure 4e, the results showed that the cutting temperature increases very slightly as feed rate increased from 0.1 mm/rev to about 0.14 mm/rev after which the temperature slowly decreased, for all depths of cuts tested. There was only a slight increase in temperature with depth of cut, Figure 4f, although the cutting temperature shows a sharp jump as feed rate decreased from 0.2 to 0.15 mm/rev, followed by a much smaller jump as feed rate decreased further from 0.15 to 0.1 mm/rev. For HSM, the same pattern was observed for depth of cut and feed rate, Figure 5e,f.

ANOVA analysis for conventional machining showed that the most significant parameter affecting cutting temperature was feed rate (*p*-value = 8.75 × 10^−9^), followed by cutting speed (2.05 × 10^−4^), and depth of cut (7.11 × 10^−4^). ANOVA analysis for HSM showed that the most significant parameter on cutting temperature was cutting speed (*p*-value = 7.605 × 10^−7^), followed by feed rate (*p*-value = 2.205 × 10^−5^), with depth of cut having only a slight effect (*p*-value = 0.037).

By comparing the results obtained by conventional machining and HSM, it was concluded that the same approximate trends existed between process parameters and cutting temperature for both cases. The difference between the results obtained in the two cases was due to the interactions between the feed rate and cutting speed and their effect on cutting temperature, in particular, the time of contact between the tool and workpiece. In this paper, for HSM, the feed rates were substantially higher than those chosen for conventional machining, and this means the feed rate had less influence compared with conventional machining as confirmed by the ANOVA analysis. This reflects that low feed rates increased cutting temperature because the cutting insert is in contact with the workpiece for longer. Therefore, to prevent higher temperature generation during machining, it is recommended to avoid very low feed rates combined with high cutting speeds. It was also found that depth of cut had a considerable effect on cutting temperature in the case of conventional machining, but only a slight effect in the case of HSM. Therefore, it is possible to use a relative higher depth of cut with high-speed turning, and thus to increase MRR, without significant additional heat generation.

**Figure 6:**
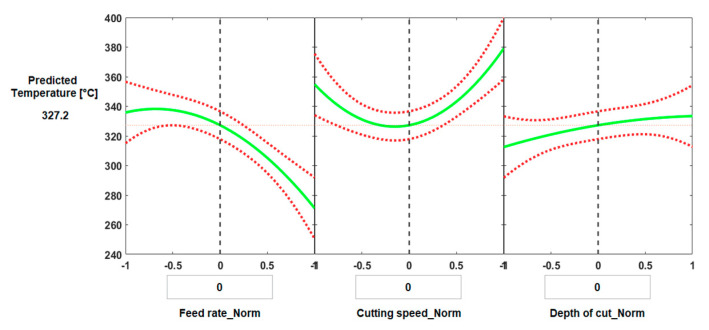
Prediction slice plots created using the developed process regression model for cutting temperature of Ti6Al4V specimens conventionally machined.

**Figure 7:**
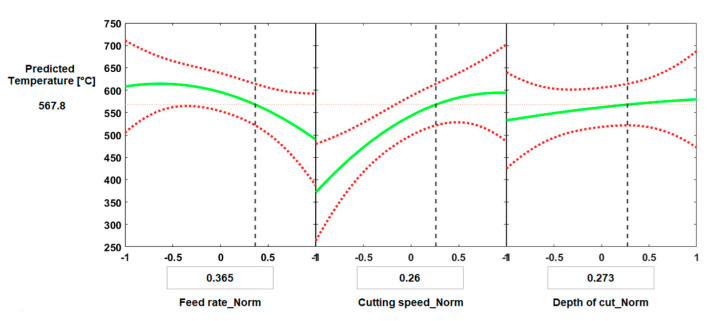
Prediction slice plots created using the developed process regression model for cutting temperature of Ti6Al4V specimens using high-speed machining.

The regression model was used in MATLAB to generate prediction plots, Figure 6 and Figure 7. Predicted slice plots are presented to show the main effects of each individual process parameter when the other parameters were kept constant. Figure 6 shows the effect of process parameters for conventional machining, where the constant values were the average values of feed rate, cutting speed and depth of cut. The green line in each plot shows the prediction in the response for the change in the normalized value of the process parameters. The dashed red curves show the 95% confidence bounds for the predicted response value. To obtain the predicted response, the vertical dashed line can move along the trends to the corresponding parameter values. This can be used to obtain those process parameters that give the minimum or maximum values of cutting temperature. Figure 7 shows how to control process parameters in order to obtain certain temperature in the case of HSM. For the given process parameters of feed rate, 0.243 mm/rev, cutting speed, 313 m/min and depth of cut, 0.273 mm, the predicted cutting temperature is 567.82 °C. This can also optimize the cutting conditions in order to obtain the minimal value of temperature and predict process parameters required to obtain any value of cutting temperature.

Experiments were carried out to test the model for different values of the cutting parameters as shown in Table 5 and Table 6. The predicted cutting temperature and measured values were determined to have an average error of (3.8%) for conventional machining and (5.3%) in the case of HSM.

### 3.2. Surface Roughness

**Figure 8:**
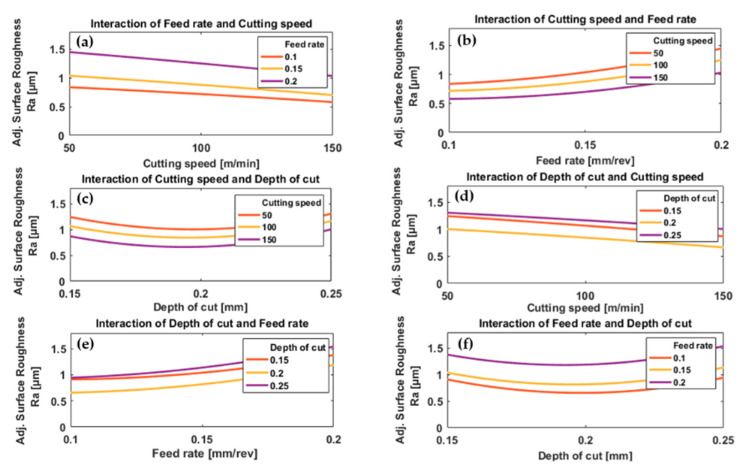
Plots depicting the interactive effects of feed rate, cutting speed, and depth of cut on surface roughness of Ti6Al4V specimens by conventional machining; (**a**) the effect of cutting speed at different levels of feed rates, (**b**) the effect of feed rate under different levels of cutting speeds, (**c**) the effect of depth of cut at different levels of cutting speeds, (**d**) the effect of cutting speed under different levels of depth of cuts, (**e**) the effect of feed rate at different levels of depth of cuts and (**f**) the effect of depth of cut under different feed rates.

**Figure 9:**
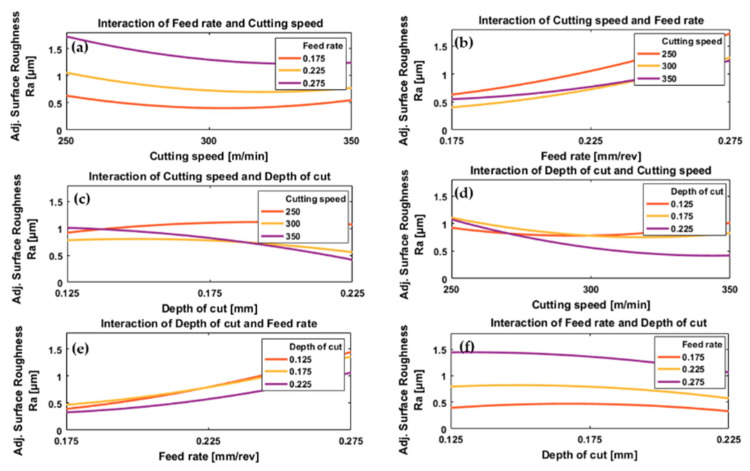
Plots depicting the interactive effects of feed rate, cutting speed, and depth of cut on surface roughness of Ti6Al4V specimens by high-speed machining; (**a**) the effect of cutting speed at different levels of feed rates, (**b**) the effect of feed rate under different levels of cutting speeds, (**c**) the effect of depth of cut at different levels of cutting speeds, (**d**) the effect of cutting speed under different levels of depth of cuts, (**e**) the effect of feed rate at different levels of depth of cuts and (**f**) the effect of depth of cut under different feed rates.

The effects of process parameters and their interactions on resulting surface roughness for conventional machining and HSM are presented in Figure 8 and Figure 9.

In the case of conventional machining, the results show a decrease in surface roughness with increasing cutting speed for all values of feed rate and depth of cut, Figure 8a,d. The reduction of surface roughness by increasing cutting speed is due to the high temperature generated by high cutting speeds which results in the softening of the workpiece material and reduced cutting force [43], and thus the fluctuations in the cutting force are smaller and result in a smoother surface. Also, with high cutting speed, there will be a reduction in the tool-chip contact length [64] and friction between tool and workpiece [65] which help to produce more uniform shearing and chip separation leading to a smoother surface. At low cutting speed, increasing friction between tool and workpiece [64] increase the possibility of build-up of edge formation resulting in poorer surface finishing [52]. These results agree with [26]. In the case of HSM, Figure 9a,d, increasing cutting speed initially decreased surface roughness slightly, then as cutting speed was increased further there was a small increase in surface roughness. The increase in surface roughness at the higher cutting speeds is due to “chatter”, a vibrational phenomenon occurring at higher values of cutting speed [67].

Increasing the feed rate gradually accelerated the increase in surface roughness, for a given cutting speed and depth of cut, Figure 8b,e and Figure 9b,e. A low feed rate results in improved surface roughness. This result agrees with most previous research that a lower feed rate gives a smoother machined surface [40].

In the case of conventional machining, surface roughness as a function of depth of cut showed a shallow “U”-shaped curve for all feed rates and cutting speeds, Figure 8c,f. The minimum was consistently at a depth of cut of 0.2 mm. The increasing surface roughness at lower depths of cut is mainly due to the large nose radius of the cutting insert used in these experiments because, at shallower depths of cut, the material ploughed rather than forming chips resulting in poor surface roughness as mentioned in [68]. However, in the case of larger depth of cut, there are benefits of having a large nose radius for the cutting insert such as improved surface roughness [26]. This phenomenon is important when machining titanium alloys due to their low modulus of elasticity which encourages elastic recovery [29]. The effect of ploughing and rubbing actions decrease with the increase in depth of cut and, as a result, surface roughness tends to decrease with increasing depth of cut, see Figure 8c,f. After passing through a minimum value (here at 0.2 mm) the surface roughness increases with a further increase in cutting depth. This is due to “chatter” as mentioned earlier [67]. In the case of HSM, Figure 9c,f, the effect of depth of cut on surface roughness showed a different trend from conventional machining, an inverted “U” shape where the maximum value depended on cutting speed and feed rate. Generally, surface roughness increased with decreasing depth of cut, Figure 9f, which is the opposite of the trend observed with conventional machining. Please note that the range of applied depth of cuts used in HSM is relatively smaller than those used in conventional machining.

By inspection of Figure 9a we see that for HSM the plot of surface roughness against cutting speed has a “U” shape with a minimum that depended on feed rate. However, we also see that the difference in surface roughness due to the changes in feed rate (0.175 to 0.275 mm/rev) was much greater than the difference due to the change in cutting speed (250 to 350 m/min), see Figure 9a,b. At low feed rates, Figure 9b, it was found that surface roughness was greater for the highest cutting speed (350 m/min) than for the lower cutting speed of 300 m/min. This slight increase in surface roughness was attributed to chatter which, for high-speed cutting dominates in the case of a low feed rate [62].

In HSM, Figure 9c an inverse relationship was found between surface roughness and depth of cut at higher cutting speeds (350 and 300 m/min) while an opposite trend was found at a cutting speed of 250 m/min. However, surface roughness was less influenced by cutting speed at lower depths of cut. At the lowest cutting speed (250 m/min), the smallest depth of cut gave the smoothest surface, but at the highest cutting speed (350 m/min) it gave the roughest surface, Figure 9d. Inversely, at the lowest cutting speed, the greatest depth of cut gave the roughest surface, but at the highest cutting speed it gave the smoothest surface; this is obviously different from the results obtained with conventional machining and due to the softening of material at high cutting speeds due to increased temperature, which reduced the cutting force required and the vibration generated during machining at greater depth of cut, and results in a smoother surface. This explains the improvement in surface roughness when machining with a high cutting speed (350 m/min) and depth of cut (0.225 mm), Figure 9d.

With conventional machining, the surface roughness increases uniformly with feed rate for all depths of cut, Figure 8e, but exhibits a shallow “U” shape when plotted against depth of cut, with a minimum at about 0.2 mm, for all feed rates, Figure 8f. No significant effect due to interaction between feed rate and cutting speed was observed. For HSM, Figure 9e it is observed that surface roughness increased with feed rate for all depths of cut but that at low feed rates the minimum depth of cut did not produce minimum surface roughness, unlike at the highest feed rates. In Figure 9f we see that the plot of surface roughness against depth of cut is a shallow inverted “U” shape with a maximum that depends on feed rate. Here, surface roughness is influenced relatively more by feed rate than cutting depth, as a greater increase in surface roughness was found at lowest depth of cut (0.125 mm) with highest feed rate (0.275 mm/rev).

ANOVA showed the most significant parameter affecting surface roughness in both cases of machining was feed rate, with (*p*-value = 1.23 × 10^−4^) for conventional machining and (*p*-value = 1.991 × 10^−7^) for HSM; the second most significant parameter was cutting speed with (*p*-value = 0.0042) for conventional machining and (*p*-value = 0.0046) for HSM; while depth of cut was third with only a slight effect on surface roughness with (*p*-value = 0.3265) for conventional machining and (*p*-value = 0.01814) for HSM.

**Figure 10:**
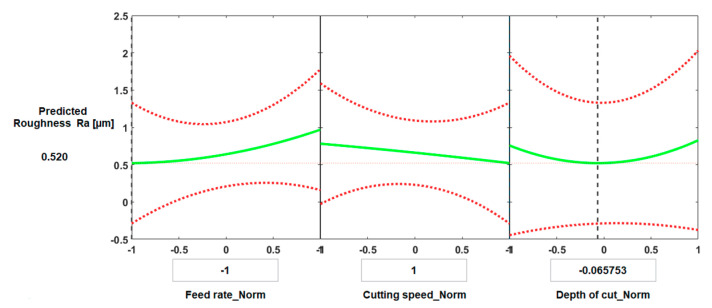
Prediction slice plots created using the developed process regression model for surface roughness of Ti6Al4V specimens with conventional machining.

**Figure 11:**
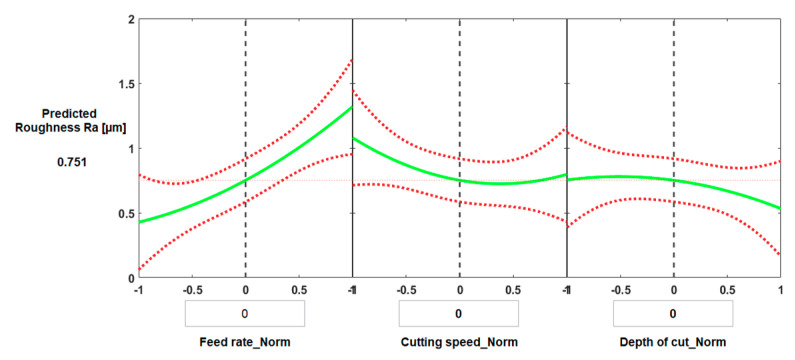
Prediction slice plots created using the developed process regression model for surface roughness of Ti6Al4V specimens machined at high speed.

Figure 10 and Figure 11 present the predictive graphs for both cases of machining. Figure 10 presents the predicted results for the optimal value of surface roughness for conventional machining. The predicted minimum surface roughness was 0.520 µm, and the corresponding values of the process parameters were: feed rate = 0.1 mm/rev, cutting speed = 150 m/min, and depth of cut = 0.196 mm.

Figure 11 shows the main effect of process parameters on surface roughness for HSM when all process parameters have average values. The predicted surface roughness was 0.751 µm, and the corresponding normalized values of the process parameters were: feed rate = 0.225 mm/rev, cutting speed = 300 m/min, and depth of cut = 0.175 mm.

Experiments were carried out to validate the theoretical model predicting surface roughness. As shown in Table 7 and Table 8, the predicted values of surface roughness and measured values were determined to have an average error of (8.5%) in the case of conventional machining, and an error of (10.4%) in the case of HSM.

**Figure 12:**
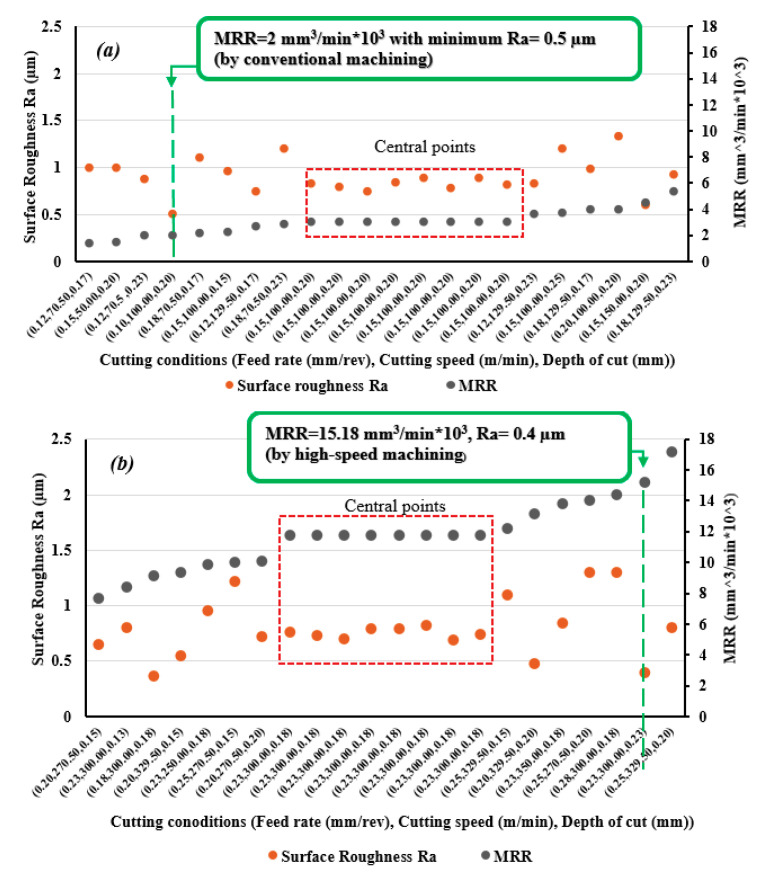
Measured Ra and calculated metal removal rate (MRR) for, (**a**) conventional machining, and (**b**) high-speed machining of Ti6Al14V.

To compare performances of conventional and high-speed machining in term of surface roughness and MRR, measured values of surface roughness and calculated values of MRR are presented in Figure 12a,b, for each trial. It was found that HSM gave good results for surface roughness compared with conventional machining, with an average improvement of 11.6%. Also, HSM achieved 207% average increase in MRR. These results show better performance for turning Ti6Al4V in terms of improving surface roughness and increasing productivity. HSM can attain a high MRR reaching 15.2 × 10^3^ mm^3^/min with a smoother surface of Ra = 0.4 µm, see Figure 10 for cutting conditions (feed rate = 0.225 mm/rev, cutting speed = 300 m/min, and depth of cut = 0.225 mm); whereas, in the case of conventional machining, minimum surface roughness of Ra = 0.5 µm was achieved for cutting parameters (feed rate = 0.10 mm/rev, cutting speed = 100 m/min, and depth of cut = 0.20 mm) that lead to a low MRR of 2 × 10^3^ mm^3^/min.

### 3.3. Relationship between Cutting Temperature and Surface Roughness

**Figure 13:**
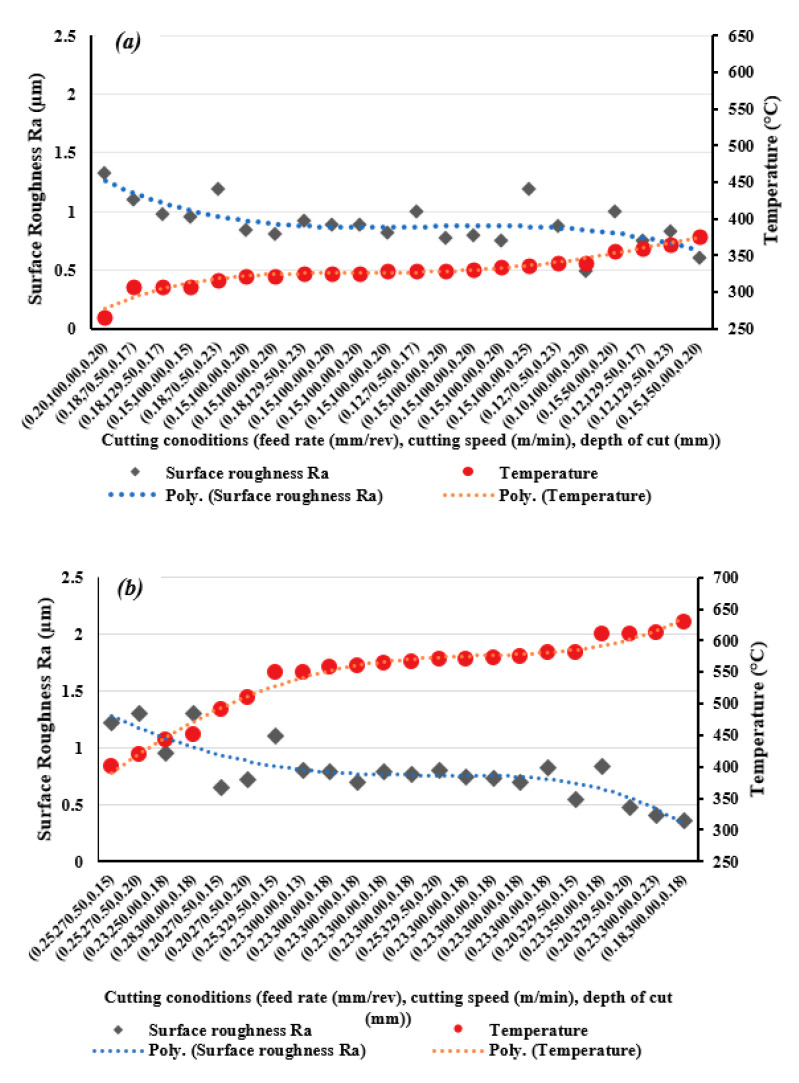
Material removal rate (MRR) and surface roughness (Ra) of Ti6Al4V specimens (**a**) by conventional machining and (**b**) by high-speed machining.

Figure 13a,b, shows all results for measured surface roughness and corresponding cutting temperatures for each trial for both conventional machining and HSM. In each case an inverse relationship between cutting temperature and surface roughness was found. This confirms the presence of an effect on cutting temperature as a result of varying cutting parameters to minimize surface roughness. This phenomenon is also mentioned in [69] where it was found when turning AISI 4340. Similarly, due to the high strength of titanium alloys, high cutting forces are required and increasing cutting temperature helps soften pre-cut material and decrease yield strength and hardness. The low thermal conductivity of titanium helps in softening the material by retaining the heat generated in the cutting zone. This can result in a reduction in cutting force [43] and makes the cutting process easier producing a smoother surface. This confirms the findings of previous studies which have reported that the mechanism of cutting of titanium-based alloys occurs by adiabatic shear band formation due to high strain rates occurring at high cutting temperatures, and that in HSM a large amount of strain occurs in the shear plane within a very short period of time [45,67]. It has been observed that improved machinability of titanium alloys has been obtained by using an external heat source such as a laser [70].

Looking at the two graphs in Figure 13, it can be seen that improvement in surface roughness by increasing temperature is more obvious in the case of HSM than conventional machining. This is because the cutting temperature generated correlates well with the temperature range which is effective in softening the material [44]. The lower cutting temperatures observed in the case of conventional machining were not sufficient to influence surface roughness.

When comparing the two sets of graphs in Figure 13, note that the conventional machining was conducted using considerably lower feed rates than those used in HSM, and feed rate has a most significant effect on surface roughness. However, HSM machining gave better results for surface roughness and it is concluded that HSM is an effective method for improving surface roughness when machining titanium alloys. HSM also offers higher MRR values, thus higher productivity. Cutting temperature obviously increased in the case of HSM and this had a positive effect in softening the Ti6Al4V material and improving performance in terms of surface roughness.

However, increasing cutting temperature during machining is not welcome as it has a negative effect on tool life. Therefore, it is recommended to use a high feed rate with HSM to avoid increasing the temperature on the tool rake face due to a low feed rate. Based on the results obtained for the two types of machining, it can be said that HSM is a successful technique for machining Ti6Al4V by PCD.

## 4. Conclusions

Comparison of machining performance between HSM and conventional machining of Ti6Al4V based on experiment and statistical analysis have been carried out. Experiments were conducted using PCD inserts with varying process parameters: cutting speed, feed rate and depth of cut. Cutting temperature, surface roughness and MRR were measured as machining outputs. The main conclusions are as follows.

- High-speed machining showed greater increases in cutting temperatures, by an average of 65% compared with conventional machining. With both machining processes, cutting speed and feed rate had a significant impact on cutting temperature and on surface roughness.
- For both machining processes a reduction in cutting temperature was observed with increasing feed rate. The effect of depth of cut on cutting temperature was found to be negligible for high-speed machining, but significant for conventional machining.
- Higher depth of cut slightly improved surface quality in the case of HSM. Accordingly, it is possible to use a relatively higher depth of cut in high-speed turning, and thus to increase MRR, in order to obtain a smoother surface without a significant effect in terms of cutting heat generation.
- Experimentally a relationship was found between generated cutting temperature and measured surface roughness. Increasing cutting temperature was found to improve surface roughness, and this has a much stronger effect in the case of high-speed machining compared to conventional machining.
- High-speed machining showed a better performance when turning Ti6Al4V compared with conventional machining in terms of improved surface roughness (although, when using higher feed rates, the results compared to conventional machining) and increased MRR, thus offering increased productivity. Increasing cutting temperature by the use of high-speed machining has the benefit of decreasing surface roughness. However, to avoid undesirable extreme temperatures, it is recommended to avoid using low feed rates.
- Finally, high cutting temperature generated by high-speed machining improved surface roughness, but this paper did not address the effect on tool wear. Future work could determine the critical range of cutting temperatures that maintains machining reliability with less effect on tool life, while improving machining output.

## Figures and Tables

**Table 1:** Chemical composition of Ti6Al4V.

Element	Ti	Al	V	Cu	Fe	Sn	Si	W
%	89.4	5.74	4.4	0.069	0.162	0.014	0.0119	0.165

**Table 2:** Cutting conditions used in high-speed machining.

High-Speed Machining			
Normalized values	Feed rate (f) mm/rev	Cutting speed (v_c_) m/min	Depth of cut (a_p_) mm
−1	0.175	250	0.125
−0.59	0.1955	270.5	0.1455
0	0.225	300	0.175
0.59	0.2545	329.5	0.2045
1	0.275	350	0.225

**Table 3:** Cutting conditions used in conventional machining.

Conventional Machining			
normalized values	Feed rate (f) mm/rev	Cutting speed (v_c_) m/min	Depth of cut (a_p_) mm
−1	0.1	50	0.15
−0.59	0.1205	70.5	0.1705
0	0.15	100	0.2
0.59	0.1795	129.5	0.2295
1	0.2	150	0.25

**Table 4:** Design of experiments.

Trial Set Index	Normalized Value (f)	Normalized Value (vc)	Normalized Value (ap)
1	0.59	−0.59	−0.59
2	0.59	0.59	−0.59
3	−0.59	0.59	0.59
4	−0.59	−0.59	−0.59
5	−1	0	0
6	0	0	1
7	−0.59	−0.59	0.59
8	0	−1	0
9	0.59	−0.59	0.59
10	0	0	−1
11	−0.59	0.59	−0.59
12	0	1	0
13	0.59	0.59	0.59
14	1	0	0
15–22	0	0	0

**Table 5:** Validation results: measured vs. predicted cutting temperature using conventional machining.

#	Feed Rate (mm/rev)	Cutting Speed (m/min)	Depth of Cut (mm)	Cutting Temperature (°C)		
				Measured	Predicted	Error %
1	0.1	150	0.195	413.5	403.7	2.4
2	0.165	90	0.18	323.0	310.3	3.9
3	0.137	62.5	0.237	331.0	348.5	−5.2
	Average of absolute error %					3.8

**Table 6:** Validation results: measured vs. predicted cutting temperature using high-speed machining.

#	Feed Rate (mm/rev)	Cutting Speed (m/min)	Depth of Cut (mm)	Cutting Temperature (°C)		
				Measured	Predicted	Error %
1	0.243	313	0.188	590.1	567.8	3.7
2	0.209	284.2	0.162	522.0	546.2	−4.6
3	0.195	340	0.205	680.0	627.0	7.7
	Average of absolute error %					5.3

**Table 7:** Validation results: measured vs. predicted surface roughness by conventional machining.

#	Feed Rate (mm/rev)	Cutting Speed (m/min)	Depth of Cut (mm)	Surface Roughness (µm)		
				Measured	Predicted	Error %
1	0.1	150	0.195	0.57	0.52	8.7
2	0.165	90	0.18	1.1	0.98	10.9
3	0.137	62.5	0.237	0.98	1.04	−6.1
	Average of absolute error %					8.5

**Table 8:** Validation results: measured vs. predicted surface roughness by high-speed machining.

#	Feed Rate (mm/rev)	Cutting Speed (m/min)	Depth of Cut (mm)	Surface Roughness (µm)		
				Measured	Predicted	Error %
1	0.243	313	0.188	0.77	0.837	−8.7
2	0.209	284.2	0.162	0.76	0.686	9.7
3	0.195	340	0.205	0.47	0.409	12.9
	Average of absolute error %					10.4
